# Overlap in the cortical representation of hand and forearm muscles as assessed by navigated TMS

**DOI:** 10.1016/j.ynirp.2023.100183

**Published:** 2023-09-05

**Authors:** Fang Jin, Sjoerd M. Bruijn, Andreas Daffertshofer

**Affiliations:** a Department of Human Movement Sciences, Faculty of Behavioural and Movement Sciences, Vrije Universiteit Amsterdam, Amsterdam, Netherlands; b Institute Brain and Behavior Amsterdam, Vrije Universiteit Amsterdam, Amsterdam, Netherlands

**Keywords:** TMS, Neuro-navigation, Motor mapping, Cortical overlap, Muscle synergies

## Abstract

The representation of upper limb muscles in the motor cortex is not clear-cut. The motor cortex contains areas that, when stimulated, may activate different muscles simultaneously, hence they seem to overlap. We expected the cortical representations of synergistic muscle pairs to overlap more than those of non-synergistic muscles. To test this, we used navigated transcranial magnetic stimulation to probe eight hand and forearm muscles of twenty healthy participants. We transformed the cortical representations of muscles to a template MRI to allow for group analysis. We found that the amount of overlap in cortical representations differed significantly between within-hand and within-forearm muscle combinations. Most synergistic muscle pairs, both within the hand, within the forearm and between them, had a larger overlap than non-synergistic muscle pairs. Our study demonstrates the largely overlapping nature of cortical representations of upper limb muscles. It is noteworthy that the overlap is elevated in muscles that usually act in a synergistic manner.

## Highlights

• We assessed overlap of cortical representations for upper extremity muscles using navigated TMS.
• Subjects' stimulation points were warped to the MNI template to allow for group analysis.
• Muscle pairs that form synergies had more overlap than of non-synergistic muscle pairs.

## 1 Introduction

Penfield and Boldrey (1937) localised the somatotopic representation of motor processing in the primary motor cortex (M1) using direct electrical stimulation of the cortex in man. While many subsequent studies established the principal structure of this motor homunculus, more fine-grained approaches revealed that it may not have a well-ordered organisation, in that especially upper limb muscles can have complex and sometimes overlapping representations (Schieber, 2001). Shinoda, Yokota, and Futami (Shinoda et al., 1981) demonstrated that axon collaterals from a single corticospinal neuron branch into motor neuron pools of at least four muscles. More recent imaging studies using fMRI (Plow et al., 2010; Huber et al., 2020) or transcranial magnetic stimulation (TMS) (van Elswijk et al., 2008; Schabrun et al., 2009; Raffin and Siebner, 2019; Sigurdsson et al., 2020) confirmed that cortical representations of distinct upper limb muscles may overlap. A very recent study by Gordon et al. (2023) showed that the “classic homunculus is interrupted by regions with distinct connectivity, structure and function, alternating with effector-specific (foot, hand and mouth) areas” (p. 351) which suggests that overlap in cortical representation of muscles may indicate synergistic control of muscles (Massé‐Alarie et al., 2017). The functional relevance of such overlap in cortical representation was underscored by Tyč and Boyadjian (2011), who found an increase in the overlap between distal and proximal upper limb muscles due to motor training. Interestingly, different motor pathologies are characterized by an increased overlap of cortical representations, not necessarily restricted to the upper extremities (Yao et al., 2009; Tsao et al., 2011; Schabrun et al., 2014; Marneweck et al., 2018). According to Yao, Chen, Carmona, and Dewald (2009), for instance, chronic stroke may result in an overlap in elbow and shoulder cortical representations, and such overlap is associated with the loss of independent control of elbow and shoulder motions; a hallmark for compensatory strategies in stroke survivors. And Elgueta‐Cancino et al. (2021) reported the overlap between deep and superficial fibres of the multifidus muscle to be correlated with the (experienced) severity of low back pain.

As a non-invasive technique, TMS is well suited to investigate whether, and to what extent, cortical representation of muscles overlap. For instance, Melgari et al. (2008) employed TMS to assess the amount of overlap between twelve upper limb muscles. They found a more pronounced overlap of hand-hand and forearm-forearm muscles combinations than between hand and forearm muscles. More recently, Tardelli et al. (2022) reported representations of forearm muscles to overlap more than those of intrinsic hand muscles. Muscles that are typically co-activated during movement seem to have more cortical overlap than others (Tardelli et al., 2022). Especially synergistic muscles seem to overlap more than non-synergistic ones (Massé‐Alarie et al., 2017).

All the studies investigating overlap in cortical representation of muscles using TMS have analysed data in subject-specific MRI coordinates. Doing so may come with substantial variability in the positioning of the TMS coil relative to functionally relevant parts of the cortex because brain morphology can vary greatly between subjects. This variability may be problematic for group-based analyses. In the current study we sought to limit this variability by warping the subject-specific data to the common MNI template. We employed this approach in a group of participants to further unravel the signature of synergistic muscle combinations. For this, we investigated the cortical representations of eight hand and forearm muscles in twenty healthy volunteers. Navigated single-pulse TMS with a pseudo-random TMS positioning (Van De Ruit et al., 2015) served to elicit motor-evoked potentials (MEPs) that we used as muscle-specific measure of excitability. Per muscle we analysed the areas spanned by the points at which MEPs were elicited on subject-specific cortical surfaces (Jin et al., 2022; Krieg, 2017), which we then transformed to the aforementioned template for group analysis. We show the muscles to have distinct, albeit partially overlapping cortical representations consistently when projected on the MNI pial surface. We further show that the overlaps between hand-hand and forearm-forearm versus hand-forearm muscle pairings differ and that synergistic muscles overlap more than non-synergistic ones.

## 2 Methods

The data used in this study have been published (Jin et al., 2022) together with a detailed description of the experimental procedures and (motivation of our) data pre-processing. There we tested the reliability of the experimental assessments and consistency of findings when estimating muscle-specific areas of excitability on individual cortex surfaces.

In brief, twenty healthy adults (eight females) participated in the study. Prior to the experimental assessments they filled out standard TMS and MRI screening questionnaires and were informed of the measurement procedures and potential risks. All participants provided signed informed consent. The study had been approved by VUmc Medical Ethics Committee (2018.213 - NL65023.029.18). The experiment was conducted in line with the Declaration of Helsinki.

### 2.1 TMS measurement

Prior to TMS application, all the participants underwent T1-weighted MRI scanning (3T Achieva, Philips, Best, The Netherlands; matrix size 256 × 256 × 211, voxel size 1.0 × 1.0×1.0 mm^3^, and TR/TE 6.40/2.94 ms). We integrated the anatomical scans in the neuro-navigation system (Neural Navigator, Brain Science Tools BV, De Bilt, The Netherlands, www.brainsciencetools.com) by segmenting them for grey matter using SPM (SPM12, https://www.fil.ion.ucl.ac.uk/spm/software/spm12/) and identifying four fiducial points (nasion, nose tip, left and right peri-auricular points) for co-registration.

**Fig. 1:**
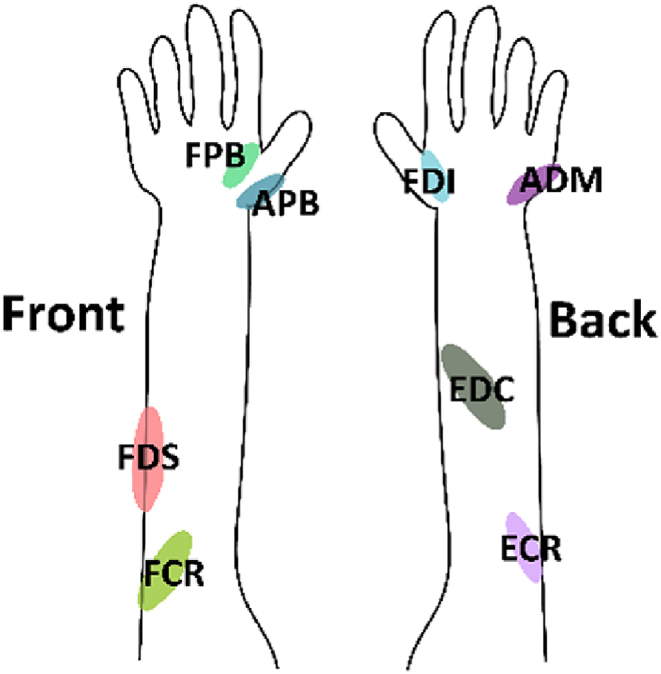
Eight muscles of the right hand and right forearm considered in this study: first dorsal interosseous (FDI), abductor digiti minimi (ADM), flexor pollicis brevis (FPB), abductor pollicis brevis (APB), extensor digitorum communis (EDC), flexor digitorum superficialis (FDS), extensor carpi radialis (ECR), and flexor carpi radialis (FCR). In the left panel the palm is pointing upward, in the right panel it faces downward.

Single-pulse mono-phasic stimulations were delivered using a Magstim 200^2^ TMS stimulator with a 70 mm diameter figure-of-eight coil (Magstim Company Ltd., Whitland, Dyfed, UK). The elicited MEPs were captured by a 16-channel EMG amplifier (Porti, TMSi, Oldenzaal, the Netherlands) and sampled at 2 kHz. Bipolar electrodes were positioned following SENIAM convention; see Fig. 1.

After identifying the respective hot spots, we determined the resting motor thresholds (RMTs) for FDI, EDC, and FCR. In brief, for each of the three muscles we first applied stimulations with increasing intensity until a consistent MEP appeared. Then, we applied thirty stimulations at around central gyrus (M1), from which the point with the highest MEP amplitude was considered as hot spot. At the hot spot, the RMT was determined as the lowest intensity at which the MEP amplitude exceeded 50 μV in five out of ten stimulations. The three RMTs served to define three conditions, namely stimulation intensities set to 105% of the respective RMTs. We deliberately chose such low stimulation intensities to limit the affected cortical region (Krieg, 2017) and, by doing so, to avoid spurious overlap between cortical representations at higher intensities (Tardelli et al., 2022; Jin et al., 2022).

Per intensity we stimulated 120 times with a 5s inter-stimulation duration (fixed via a revised version of https://github.com/armanabraham/Rapid2). We repeated this procedure once, yielding at total of 3 (intensities) × 120 (stimulations) × 2 (repetitions) = 720 stimulations per participant. As already mentioned, we employed a pseudo-random coil positioning (Van De Ruit et al., 2015) that covered roughly 5×5 cm around the corresponding hot spot.

### 2.2 Data analysis

The EMG signals were high-pass filtered at 30 Hz using a 2nd-order bi-directional Butterworth filter. We identified stimulations with proper motor-evoked potential (MEPs) based on the peak-to-peak EMG amplitude (less than 10 mV but larger than twenty times the baseline’s standard deviation obtained 200 ms prior to stimulation; see (Jin et al., 2022) for more details). All stimulations were classified as either MEP or non-MEP points. Stimulations outside M1 (“precentral L” in the “Mindboggle6” atlas (Klein and Hirsch, 2005)) were eliminated; see also the *Supplementary Section* S7 for the analysis without this constraint.

Our data analysis relied on the individual MRIs that were segmented using FreeSurfer (http://surfer.nmr.mgh.harvard.edu/) and we used the pial surface for all subsequent steps. For the within-group comparison we used the MNI152 default subject implemented in Brainstorm (Tadel et al., 2011) as template surface; see *Supplementary Sections* S6 and S7 for the analyses on the subject-specific MRIs.

**Fig. 2:**
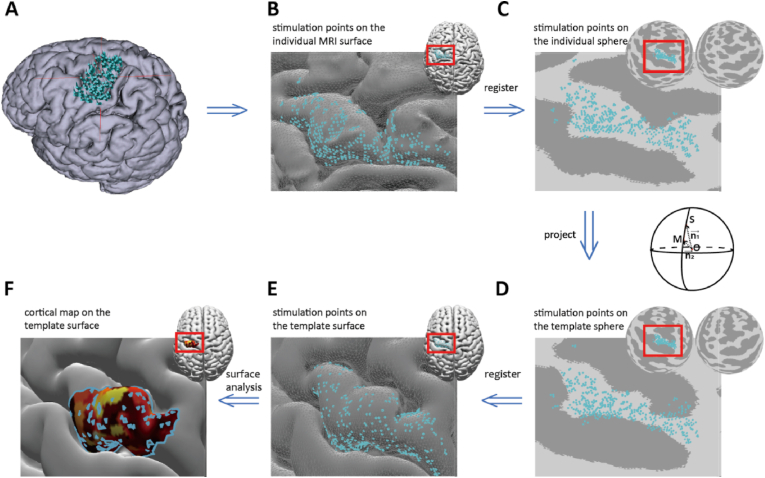
Processing pipeline. The blue dots represent the stimulation points (denoted by S on the sphere). The surfaces in panels A and B are the individual MRI surfaces, and panel C depicts the mapping to the unit spheres per hemisphere. The lower row relates to the template representation where panel D is the transform to the corresponding unit spheres that can readily be mapped to the MNI152 template (panel E). Panel F displays the estimated area of excitability, colour-coded by the MEP amplitudes. (For interpretation of the references to colour in this figure legend, the reader is referred to the Web version of this article.)

The subsequent analysis steps are illustrated in Fig. 2. Coil coordinates and orientations were registered from the individual MRI surface (panel B) and we inflated each hemisphere to a unit sphere (panel C) (Tadel et al., 2011; Saad et al., 2004). To map a stimulation to the template (panel D), we searched for the sphere-inflated template vertex by incorporating subject-specific gyri and sulci locations via their curvature values and warped to the respective inflated (spherical) template by minimising their great-circle distance. The resulting points (panel D) were finally deflated to a template surface (panel E).

We used our open-source surface analysis toolbox (https://github.com/marlow17/surfaceanalysis) (Jin et al., 2022) to quantify the cortical representation of every muscle by its area. In the triangulated cortex mesh, we first determined the area’s vertices, i.e., $A=\left\{{\vec{v}}_{1},\ldots ,{\vec{v}}_{N}\right\}$ with ${\vec{v}}_{i}{=\left(v_{x,i},v_{y,i},v_{z,i}\right)}^{T}$ and set their MEP values ${MEP}_{i}$ by interpolating the MEP amplitudes corresponding to the original stimulation points – see (Jin et al., 2022) for further details about the underlying search algorithm. Here, we abbreviate the resulting (MEP-weighted) area by $W=\left\{\left({\vec{v}}_{1},{MEP}_{1}\right),\ldots ,\left({\vec{v}}_{N},{MEP}_{N}\right)\right\}\text{.}$ In, e.g., Fig. 2 (panel F), the area is shown as colour-code patches with yellow indicating the largest MEP amplitudes, i.e., the highest degree of muscle-specific excitability.

Following (Jin et al., 2022), we first parametrised an area’s location via its centroid given by

(1) $$\vec{C}=\vec{C}\left(W\right)=\left(\sum_{\left(\vec{v},MEP\right)\in W}MEP∙\vec{v}\right)/\left(\sum_{\left(\vec{v},MEP\right)\in W}MEP\right)$$

Next, we computed the size of an area via the triangular prism. For this, consider a triangle in A .Let the lengths between its vertices be $\lambda_{1}={\left\|{\vec{v}}_{1}-{\vec{v}}_{2}\right\|}_{2}$, $\lambda_{2}={\left\|{\vec{v}}_{2}-{\vec{v}}_{3}\right\|}_{2}$ and $\lambda_{3}={\left\|{\vec{v}}_{3}-{\vec{v}}_{1}\right\|}_{2}$, where ${\left\|\cdots \right\|}_{2}$ denotes the Euclidean distance. Then, the area size W∶=‖W‖ reads

$$\left\|W\right\|=W=\sum_{k=1}^{M}{\bar{MEP}}_{k}\sqrt{\Lambda_{k}\left(\Lambda_{k}-\lambda_{1,k}\right)\left(\Lambda_{k}-\lambda_{2,k}\right)\left(\Lambda_{k}-\lambda_{3,k}\right)}$$

with

(2) $$\Lambda_{k}=\frac{1}{2}\sum_{i=1}^{3}\lambda_{i,k}$$

with ${\bar{MEP}}_{k}=\frac{1}{3}\sum_{i=1}^{3}{MEP}_{i,k}$ being the mean value of (interpolated) MEP amplitudes at the three vertices of triangle k.

Finally, we assessed the overlap of the cortical representations by combining the eight muscles into 28 distinct pairs (FDI-ADM, FDI-APB, …) and defining three groups of muscles: hand-hand, hand-forearm, and forearm-forearm. This grouping facilitated focusing on effects of (non-)synergistic muscle combinations. For instance, within the hand-hand group, FDI-APB, FDI-FPB, and APB-FPB are typically considered synergistic, and in the forearm-forearm group EDC-ECR and FDS-FCR are typically considered synergistic. In Supplementary Table S1.1 we provide a complete list of muscle pairs that we here considered as synergistic muscle combinations, and we motivate our choice by providing the corresponding references. Yet, we stress that these combinations also depend on the motor task being studied.

**Fig. 3:**
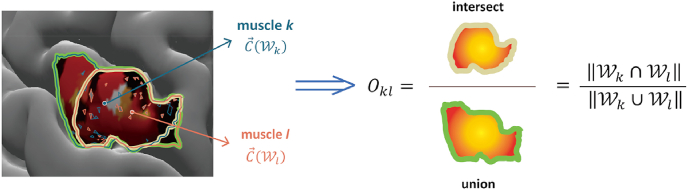
Illustration of the centroid and overlap definition of two muscles *k* and *l*. To ease visual inspection, we highlighted the contour of the two areas; cf. Eq. (3).

Evidently, the overlap O between areas can be quantified via their intersect that we here normalised using the corresponding union. That is, per muscle pair (k,l) we defined:

(3) $$O_{kl}=\frac{\left\|W_{k}\cap W_{l}\right\|}{\left\|W_{k}\cup W_{l}\right\|}$$

where ‖⋯‖ denotes the size definition given in Eq. (2). This computation is illustrated in Fig. 3. Here we would like to note that by transforming the subject-specific cortex stimulation points to the template (cf. Fig. 2) both $\vec{C}$ and $O_{kl}$ could readily enter our group analysis (see below under *Statistics*).

### 2.3 Statistics

A two-way ANOVA with factors of *Muscle* and *Intensity* as well as their interaction served to test for significant differences in the centroids $\vec{C}$ and the area size W across all muscles. Subsequently, we grouped the muscles into hand and forearm muscles and analysed between-group and within-group (hand-hand, forearm-forearm, and hand-forearm) differences in the overlaps $O_{kl}$ using a two-way ANOVA with factors *Muscle-Group* and *Intensity,* as well as their interaction. For the overlap, we also performed post-hoc tests of muscle combinations within groups of pairs. There we conducted three separate two-way ANOVAs with *Pair* and *Intensity,* as well as their interaction as factors. The results of the post-hoc analysis of all combinations are reported in tabular form in *Supplementary Section* S5. Prior to all analyses, we tested for sphericity using Mauchly’s Test and applied a Greenhouse-Geisser correction whenever deemed necessary. Throughout the analysis we considered a significant threshold of α = 0.05; all ANOVAs used repeated measures. Post-hoc assessments were Bonferroni corrected. We performed all analyses in Matlab 2022a (MathWorks, Natick, MA, USA).

Note that the number of elicited MEPs as (mean) amplitude values and the corresponding statistics have already been reported in (Jin et al., 2022); see Supplementary Table S1 and Supplementary Fig. S1 in that paper.

## 3 Results

### 3.1 Muscle representations

**Fig. 4:**
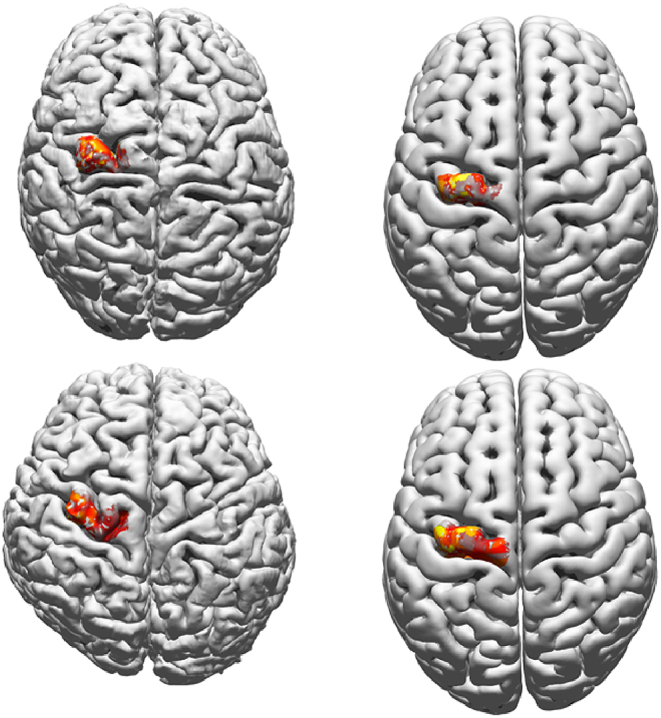
Two examples of the warping of the area of excitation of a single muscle to the template surface – left column: subject-specific cortical surface; right column: mapping onto the template surface. One participant is of European descent (upper row), the other of East-Asian descent (lower row); in both cases the MEP-weighted region of excitability of FDI has been selected. Here we would like to note that stimulation points outside the contralateral M1, defined as the left precentral area, have been removed; see Fig. 2 for the corresponding procedures. Yellow indicates large MEP-weighted value, dark red low ones; units are arbitrary due to scaling to a maximum value of 1; cf. Fig. 5. (For interpretation of the references to colour in this figure legend, the reader is referred to the Web version of this article.)

Before summarising the outcome of our hypothesis testing, we first illustrate two examples of warping the cortical surfaces to the MNI152 template surface in Fig. 4.

**Fig. 5:**
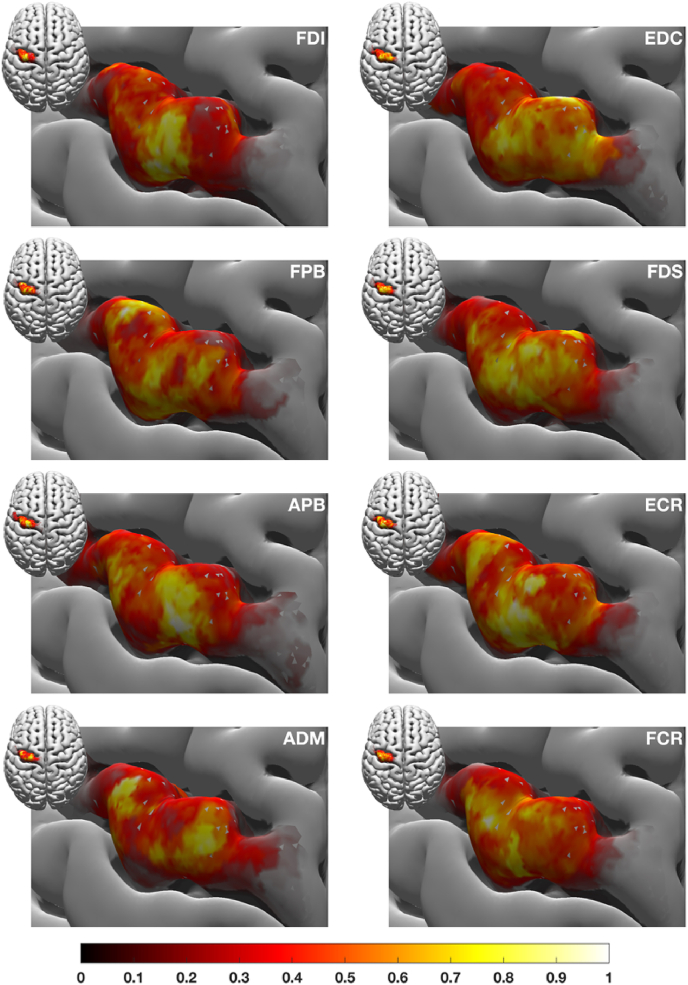
Group average of the cortical representations of the eight muscles. The areas are shown on the MNI template pial surface and represent excitability maps for the 105%-RMT-FDI intensity. The colour coding is given by the size of the corresponding MEPs, where yellow implies a large MEP amplitude and dark red a low one (the darker the colour the more transparent the plot will be to ease legibility); units are arbitrary due to scaling. The same figure with muscle-specific scaling is given in the Appendix (Figure A1). In the *Supplementary Section* S2 we show the other two stimulation intensities as well as intensity maps of some randomly selected participants (*Supplementary Section* S3). (For interpretation of the references to colour in this figure legend, the reader is referred to the Web version of this article.)

Fig. 5 depicts the group average cortical representation of the eight muscles for the 105%-RMT-FDI Intensity (= 47.25 ± 2.16% stimulator intensity; EDC = 47.68 ± 2.23% and FCR = 50.58 ± 2.28% can be found in as Supplementary Figures S2.1 and S2.2). The representation clearly differed between muscles with, on average, FDI showing the highest degree of excitability.

### 3.2 Position and size of the cortical muscle representation

**Fig. 6:**
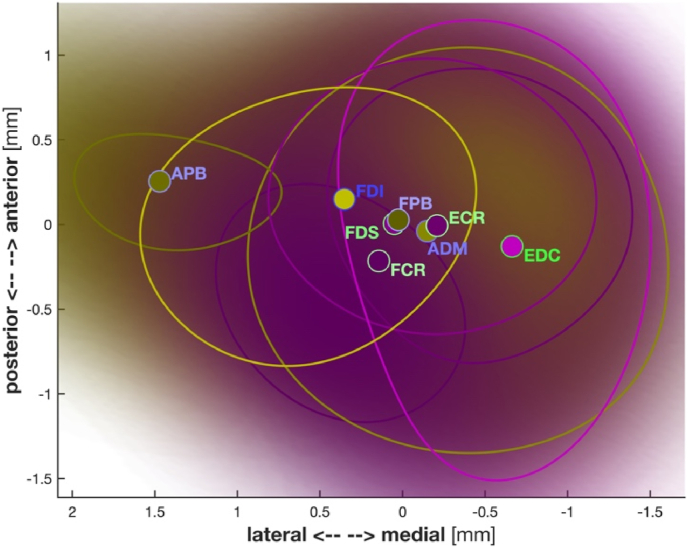
Probability densities of the centroids of the area of excitability estimated using kernel source densities over all participants. Contour lines indicate the 5% probability values. The different colours symbolise the hand (yellow) and forearm (magenta) muscles; dots are the mean centroids labelled accordingly. The hand muscles are more lateral than the forearm muscles. Note that we only depict the (anterior/posterior vs. medial/lateral direction)-plane. (For interpretation of the references to colour in this figure legend, the reader is referred to the Web version of this article.)

Overall, the hand representations were more lateral than those of the forearm muscles; see Fig. 6 where we show the average centroid positions for the 105%-RMT-FDI in the 2D-plane (anterior/posterior vs. medial/lateral direction).

As summarised in Table 1, the centroid positions in the medial/lateral and inferior/superior directions ($C_{y}$ and $C_{z}$, respectively) as well as the area size differed significantly per *Muscle*. Neither parameter showed significant effects of *Intensity* or a significant *Muscle* × *Intensity* interaction.

**Table 1:** Statistics of the cortical representation of the eight muscles. W = area size, centroid components: $C_{x}$ = medial/lateral, $C_{y}$ = anterior/posterior, $C_{z}$ = inferior/superior. Significant effects are shown in **bold.**

	Muscle		Intensity		*Muscle* × *Intensity*	
	F(7,91)	*p*	F(2,26)	*p*	F(14,182)	*p*
$C_{x}$	1.349	.273	0.354	.705	1.041	.405
$C_{y}$	4.668	**.000**	0.090	.914	1.299	.270
$C_{z}$	4.728	**.000**	0.760	.435	1.272	.288
W	3.147	**.040**	3.865	.060	1.461	.251

### 3.3 Size of the overlapping areas

**Fig. 7:**
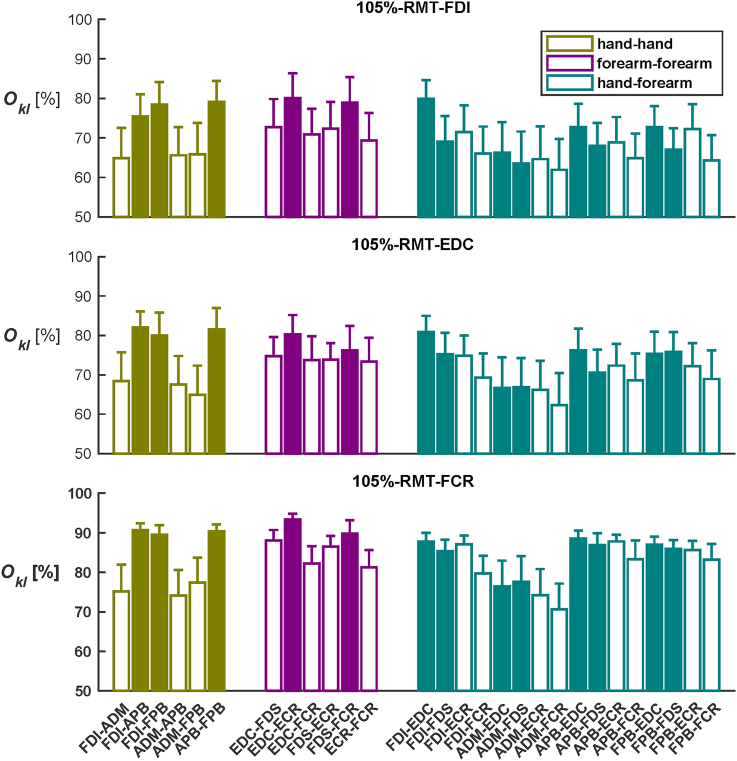
Overlap of all muscle pairs. The shown percentages of Overlaps $O_{kl}$ between muscle pairs expressed in percentage (of the union area) for the three intensities. The filled bars represent the muscle pairs with synergistic function, whereas the open bars represent the non-synergistic muscle pairs; see Supplementary Table S1.1 for a motivated definition of synergistic pairs. Error bars represent the standard error over participants. Colour coding agrees with Fig. 5. (For interpretation of the references to colour in this figure legend, the reader is referred to the Web version of this article.)

The overlap between representations differed significantly between muscle groups (F(2,38) = 208.862, *p =* .000). There was also a significant effect of *Intensity* (F(2,38) = 6.246, *p* = .005) as well as a significant *Muscle-Group* × *Intensity* interaction (F(4,76) = 5.326, *p* = .001). For the 105%-RMT-FCR Intensity, the overlap was larger than in both 105%-RMT-FDI (*p* = .021) and EDC (*p* = .011). Post-hoc pairwise comparisons revealed no significant differences in the overlap between the different pair groups. Corresponding descriptive statistics are illustrated in Fig. 7.

### 3.4 Within muscle-group overlaps (post-hoc analyses)

For the hand-hand muscle pairs the overlap displayed significant main effects of *Intensity* and *Pair* (F(2,38) = 4.979, *p* = .012 and F(5,95) = 4.021, *p* = .034, respectively) while the *Pair* × *Intensity* interaction did not reach significance (F(10,190) = 0.422, *p* = .819). The overlaps at 105%-RMT-FCR were larger than at 105%-RMT-EDC (*p* = .044). Analysing the forearm-forearm muscle group revealed comparable effects (*Intensity*: F(2,38) = 3.768, *p* = .032 and *Pair*: F(5,95) = 3.870, *p* = .016 as well as *Pair × Intensity*: F(10,190) = 0.433, *p* = .808). Again, the overlaps were larger for 105%-RMT-FCR than for 105%-RMT-EDC (*p* = .029). The overlap of the FDS-FCR combination exceeded that of ECR-FCR (*p* = .027). Finally, the overlaps of hand-forearm muscle pairs were significantly affected by the factor *Intensity* (F(2,38) = 6.987, *p* = .003) but not by *Pair* (F(15,285) = 1.859, *p* = .164). The *Pair* × *Intensity* interaction was again not significant (F(30,570) = 0.668, *p* = .706). However, here both 105%-RMT-FDI and -EDC intensities showed smaller overlaps than 105%-RMT-FCR (*p* = .012 and *p* = .013, respectively).

## 4 Discussion

We mapped eight upper limb muscles (4 hand and 4 forearm muscles) using navigated TMS and assessed the overlap in cortical representations of various muscle pairs. Using surface analysis and by warping the subject-specific cortex surfaces to a standardised template, we quantified the areas of excitability by their centroid positions and area sizes. The hand representations were more lateral, whereas those of the forearm muscles were more medial. This agrees with earlier studies that showed digit movements to be more laterally represented than upper limb movements (Schieber, 1990). We also found that the thumb muscles (FDI, APB and FPB) are lateral to the little finger muscle (ADM), as expected. Like other studies (Tardelli et al., 2022) we found the overlap at higher intensity (105% RMT of FCR) to be larger than that at relatively low intensities (105% RMT of FDI, EDC).

### 4.1 Overlap in cortical representations – synergistic versus non-synergistic muscles

Previous research in monkeys and humans demonstrated that cortical representations of the upper limb muscles can overlap (Schieber, 2001; Gordon et al., 2023; Tardelli et al., 2022). Tardelli et al. (2022) showed that the cortical representations of FCR, FPB and ADM were similar and largely overlapping. As such, we expected that also in our experimental assessments the cortical representations of the eight muscle to (partly) overlap. Yet, we hypothesised the overlap to differ between the hand-hand, forearm-forearm, and hand-forearm muscle combinations as well as between synergistic and non-synergistic muscles. As summarised in Supplementary Table S1.1., we considered FDI-APB, FDI-FPB, and APB-FPB as synergistic pairs within the hand-hand group, as they are often coupled during unimanual actions such as object manipulation. The forearm-forearm pairs EDC-ECR and FDS-FCR are usually synergistic for larger extension and flexion movements, and in the hand-forearm group, EDC-FDI, -APB, -FPB, -ADM, and FDS-FDI, -APB, -FPB, -ADM usually act in a synergistic manner. Having said that, we stress that the definition of synergistic muscle combinations is typically task specific. In fact, even the cortical representation of individual muscles may depend on the task being executed (Gooijers et al., 2022) as well as the overlap between them (Gordon et al., 2023).

The amount of overlap differed significantly between hand-hand, hand-forearm, and forearm-forearm muscle pairs groups. This is in line with Melgari et al. (2008) who showed that the amount of overlap in the hand-hand, hand-forearm, and forearm-forearm pairs was higher in hand-arm and arm-arm pairs. In addition, according to Tardelli et al. (2022), the overlap between intrinsic hand muscles (ADM-FPB) is smaller than between forearm and hand muscles (FCR-ADM and FCR-FPB). We found a significant difference between muscle-pair groups but not always so in pairwise comparisons. This discrepancy calls for future studies involving even more muscles than we currently studied, as this may clarify further how the amount of cortical overlap relates to the muscles’ anatomical locations.

By and large, the synergistic muscle pairs overlapped more than the muscle pairs without synergistic function. In the hand-hand muscle pairs, the average overlap of synergistic muscle pairs (FDI-APB, FDI-FPB, APB-FPB) was higher than the overlap in non-synergistic muscle pairs (ADM-FDI, ADM-APB, ADM-FPB). The synergistic muscle pairs (EDC-ECR, FDS-FCR) demonstrated more overlap than other forearm-forearm muscle pairs. The pairwise comparisons revealed that FDS-FCR overlapped significantly more than ECR-FCR. Massé‐Alarieet et al. (Massé‐Alarie et al., 2017) also reported the ECR-EDC overlap to be stronger than the ECR-FCR overlap, and DeJong et al. (2021) showed that the synergistic pairs FDI-APB and EDC-ECR overlap more than other muscle pairs.

The ADM-related muscles pairs (ADM-FDI, ADM-APB, and ADM-FPB) had smaller overlaps than the FDI-APB, FDI-FPB, and APB-FPB pairs. The average centroid of ADM was more medial than the centroids of FDI, APB, and FPB; see Fig. 6. We speculate that the observed overlaps between hand muscles may be associated with the distribution of the individual cortical representation. However, there was no significant difference in the centroids of cortical representation between other muscle pairs (ECR-FCR, ADM-FCR, etc.). Interestingly, Schieber (1990) observed that the neural pathways from single corticospinal neurons in primates diverged to different motoneuron pools of four muscles. We, therefore, hypothesise that the overlaps between forearm muscles resemble (the structure of) other elements (the intermediary part between corticospinal neuron to motoneuron pools) rather than cortical overlap in the motor neural pathway.

### 4.2 Standardising neuro-anatomy

Registering surfaces to the MNI template is, in principle, not needed when quantifying the overlap of cortical representation using a relative measure (in our case, the intersect divided by the union of the corresponding surface areas). When comparing the respective centroids (cf. equation (1)) or the centre-of-gravity (estimated via the points/locations of stimulations), standardising anatomy is mandatory, especially for between subject comparisons. We realise that this procedure is not yet common in TMS-studies, whereas it is in, e.g., (f)MRI. For this reason, we also conducted the same analyses on subject-specific MRIs without warping to the MNI-template. The corresponding results are reported as *Supplementary Section* S6 (in S7 we also show the case without constraining the analysis to M1). The results largely agree, at least qualitatively. This was clearly expected for our measure of the relative overlap size (see above), but also for the centroids given our within-subject statistical design. The quantitative differences stem from the fact that the surface meshes of the subject-specific MRIs and the MNI template differ in resolution (triangularisation), which cannot be circumvented. Given this large agreement we are confident that our approach to standardising anatomy is proper.

### 4.3 Limitations

In our TMS protocol we applied stimuli on average every 5s. Such a periodic stimulation train may cause changes of the MEP amplitudes due to long-term modulation of cortical excitability. To refute that this was the case in our experiment, we estimated the mean MEP-amplitude over participants and conditions as a function of time (stimulus moments). This did not reveal any clear trend in any of the muscles; see Supplementary Figure S8.1. While we cannot exclude the presence of long-term modulation of cortical excitability to all degree, we are inclined to argue that this was negligible in our study.

While random coil positioning in combination with neuro-navigation is becoming common practice, the group analysis via transferring areas of excitability on a template is novel. In fact, transforming centroid positions to the template is straightforward. Here, we used inflated spheres in combination with the great circle distance (see (Gomez-Tames et al., 2020) for an alternative, namely, the minimisation Euclidian distances in deep brain regions). While this step is common in MRI studies, its applicability depends on the quality of our surface inflation method, and this might be difficult to quantify. Yet, we believe that this positioning is more accurate than (piecewise) affine transforms using isolated anatomical landmarks. However, when warping areas (and likewise volumes), geometrical transforms locally alter the neural density rendering subsequent biophysical modelling a challenge. As such, the ‘true’ degree of excitability should be interpreted with caution.

We chose to project and represent points and areas of excitability on the MNI pial surface. Evidently, TMS does not excite the pial surface (neither is the scalp that is typically used (Melgari et al., 2008; Tardelli et al., 2022; Nazarova et al., 2021)). Yet, we decided not to project on, e.g., the grey matter segments as this would suggest that we can provide the precise location of the ‘point’ of the electric field that TMS induces, which we simply cannot (Siebner et al., 2022). Combining the here suggested warping to the template with proper estimates of the electrical field distributions (Gomez-Tames et al., 2020; Opitz et al., 2011; De Lucia et al., 2007; Weise et al., 2023) may help to solve this. Most likely this will also require analysing below the surface, i.e., one may estimate the corresponding volumes of excitability. Warping using anatomical landmarks will still be possible but may be computationally demanding.

A similar concern is that the intensity of TMS strongly influences the amount of observed overlap. Tardelli et al. (2022) and DeJong et al. (2021) already showed the relationship between the amount of overlap and intensity and noted that a higher stimulation intensity would result in a greater cortical overlap due to the non-focal characteristics of TMS. Especially at higher stimulation intensities, the observed overlap may – and probably will – be due to the stimulation at one point “radiating” to the adjacent surface, which will induce an MEP in other muscles. Any statement about the achieved spatial resolution when estimating areas (or volumes) of excitability and, more so, overlaps should therefore be questioned. To minimise this ‘leakage’ effect, we deliberately chose a minimum stimulation intensity just above RMT. We admit that we cannot exclude leakage. Estimating the electrical field distributions (Gomez-Tames et al., 2020; Opitz et al., 2011; De Lucia et al., 2007; Weise et al., 2023) may again help to solve this in the future, in particular when combined with other imaging modalities like fMRI (Vink et al., 2018). The electrical field distribution could be more informative than the focal point of the magnetic field (and its orientation that we here ignored) (Weise et al., 2020, 2023; Sollmann et al., 2016). However, estimating requires several assumptions of the corresponding bio-electrical properties, first and foremost, that of grey matter, but we certainly advocate such multi-modal approaches for future studies.

## 5 Conclusion

We used TMS to assess amount and position of the overlap between the cortical representations of eight hand and forearm muscles. We projected individual subject data to a high-resolution template cortical mesh. Hand muscles turned out to be more laterally positioned than the more medial forearm representations. Most synergistic muscle pairs displayed significantly more cortical overlap than their non-synergist counterparts.

## Declaration of competing interest

None.

## Figures and Tables

**Figure A1:**
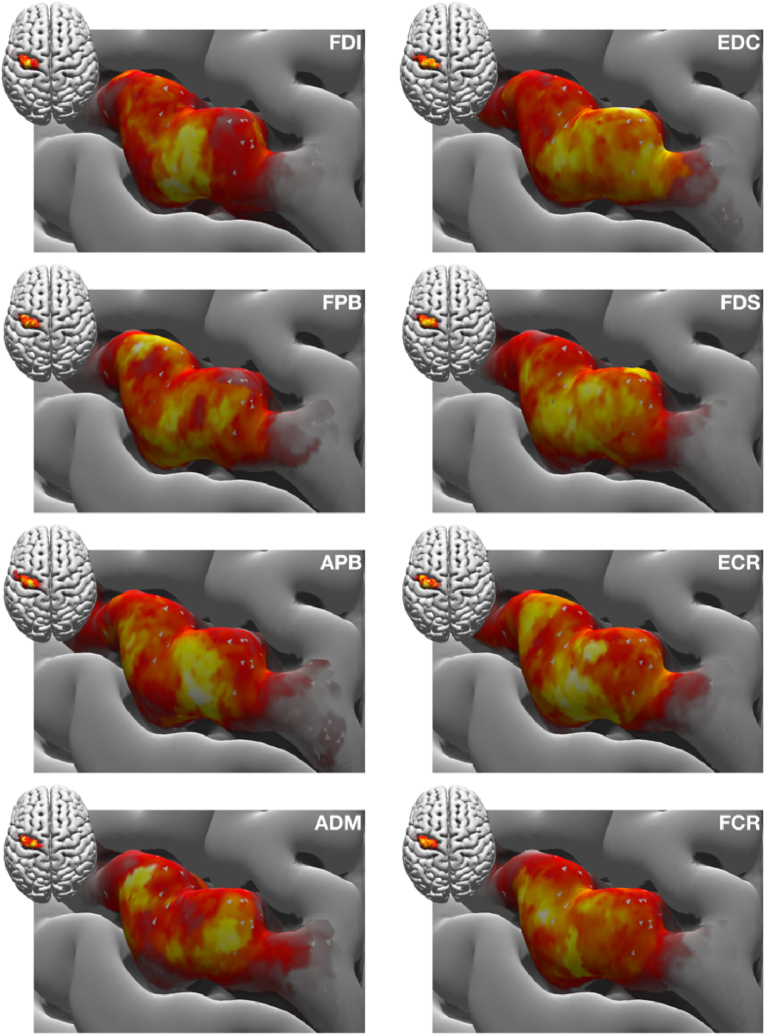
Group average of the cortical representations for the 105%-RMT-FDI Intensity. This figure agrees with Fig. 5 except for the colour-coding. While in Fig. 5 intensities (MEP amplitudes) are given on a common scale, here every muscle map is scaled separately to highlight that the cortical distributions are typically complex even when looking at isolated muscles. Note that the distributions of excitability are not unimodal. There are in fact distinct peaks of excitability rendering the representation heterogeneous is not (solely) caused by averaging over the group. The units of the colour coding are arbitrary.

## Data Availability

Data will be made available on request.
