# Supplementary material for: Overlap in the cortical representation of hand and forearm muscles as assessed by navigated TMS
Source: Neuroimage Rep. 2023 Sep 5;3(3):100183. doi: 10.1016/j.ynirp.2023.100183 (PMC12172753; doi:10.1016/j.ynirp.2023.100183)
Supplement: Multimedia component 1 [file mmc1.pdf]

S1. Synergistic muscle pairs

Table S1.1 Synergistic pairing based on the literature. Hand muscles are represented as light orange cells, and forearm muscles are represented by light blue ones. Dark orange (hand-hand), green (hand-forearm), blue (forearm-forearm) cells represent synergistic muscle pairs, the reference in the cell indicates which study suggested that that muscle pair is synergistic.

	FDI	*ADM*	*APB*	*FPB*	*EDC*	*FDS*	*ECR*	*FCR*
FDI			[1,2]	[2]	[2]	[1,2]		
ADM					[3]	[4]		
APB				[5]	[5]	[5]		
FPB						[5]		
EDC							[6]	
FDS								[7]
ECR								
FCR								

References

[1] Laine CM, Valero-Cuevas FJ. Intermuscular coherence reflects functional coordination. *Journal of Neurophysiology* 2017;118(3):1775-83.

[2] Weiss EJ, Flanders M. Muscular and postural synergies of the human hand. *Journal of Neurophysiology* 2004;92(1):523-35.

[3] Winges SA, Furuya S, Faber NJ, Flanders M. Patterns of muscle activity for digital coarticulation. *Journal of Neurophysiology* 2013;110(1):230-42.

[4] Tanzarella S, Muceli S, Santello M, Farina D. Synergistic organization of neural inputs from spinal motor neurons to extrinsic and intrinsic hand muscles. *Journal of Neuroscience* 2021;41(32):6878-91.

[5] Klein Breteler MD, Simura KJ, Flanders M. Timing of muscle activation in a hand movement sequence. *Cerebral Cortex* 2007;17(4):803-15.

[6] Lee SW, Triandafilou K, Lock BA, Kamper DG. Impairment in task-specific modulation of muscle coordination correlates with the severity of hand impairment following stroke. *PloS one* 2013;8(7):e68745.

[7] Yarossi M, Brooks DH, Erdoğmuş D, Tunik E. Similarity of hand muscle synergies elicited by transcranial magnetic stimulation and those found during voluntary movement. *Journal of Neurophysiology* 2022;128(4):994-1010.

S2. Averaged cortical representations

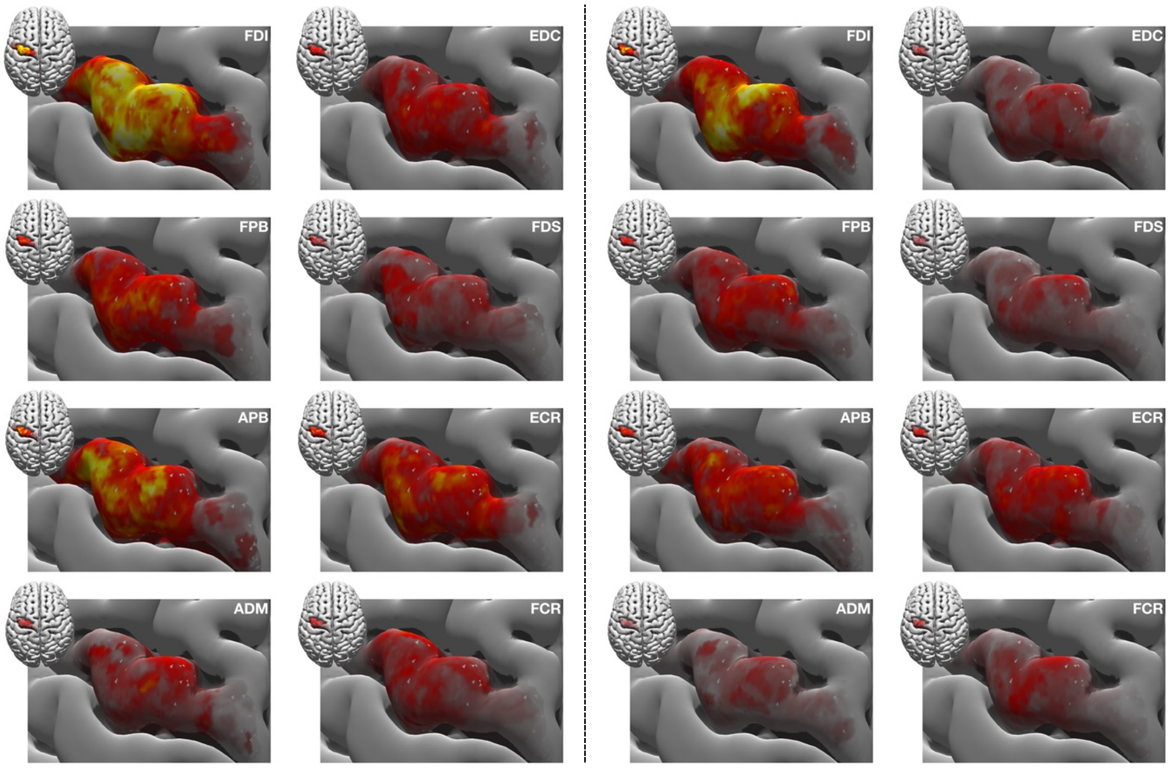

Figure S2.1 Group average of the cortical representations for the 105%-RMT-EDC and the 105%-RMT-FCR intensities (left and right columns, respectively). As in Figure 5 of the main text also here the excitability of the FDI is most pronounced.

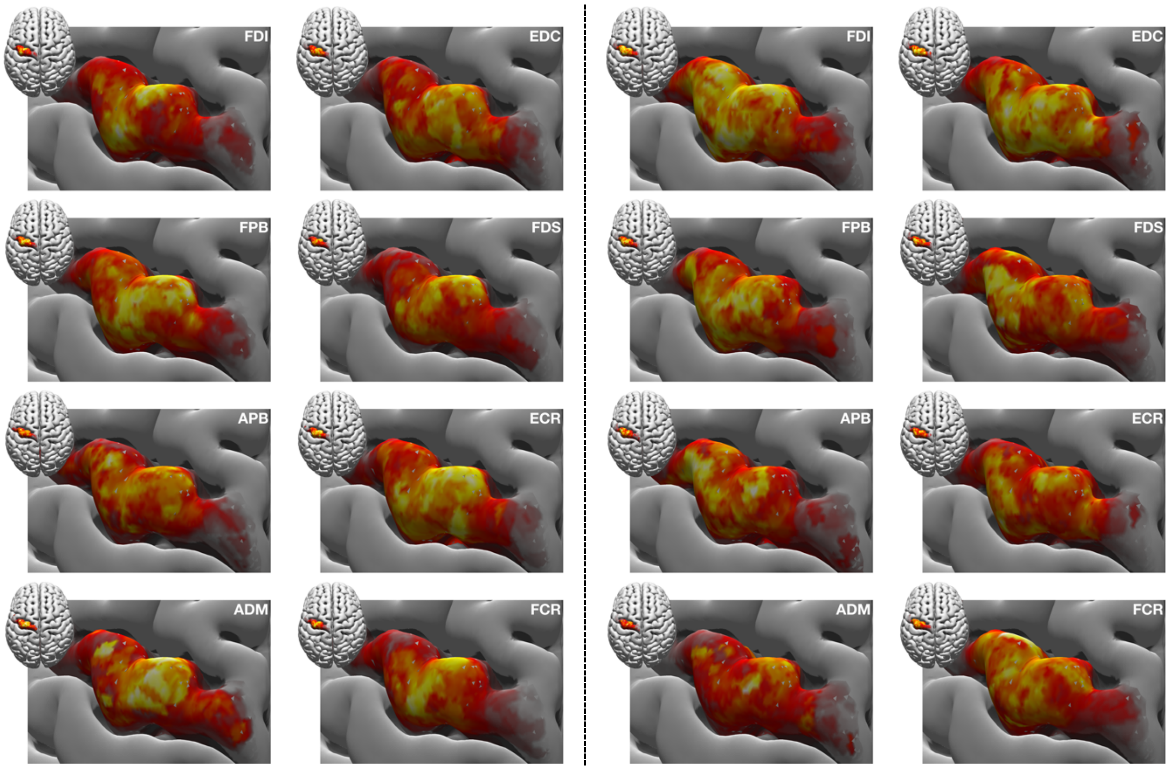

Figure S2.2 Identical to Figure S2.1 but muscle intensities are display on individual colour scales; cf. Figure A1 in the main text.

S3. Cortical representations of randomly selected subjects

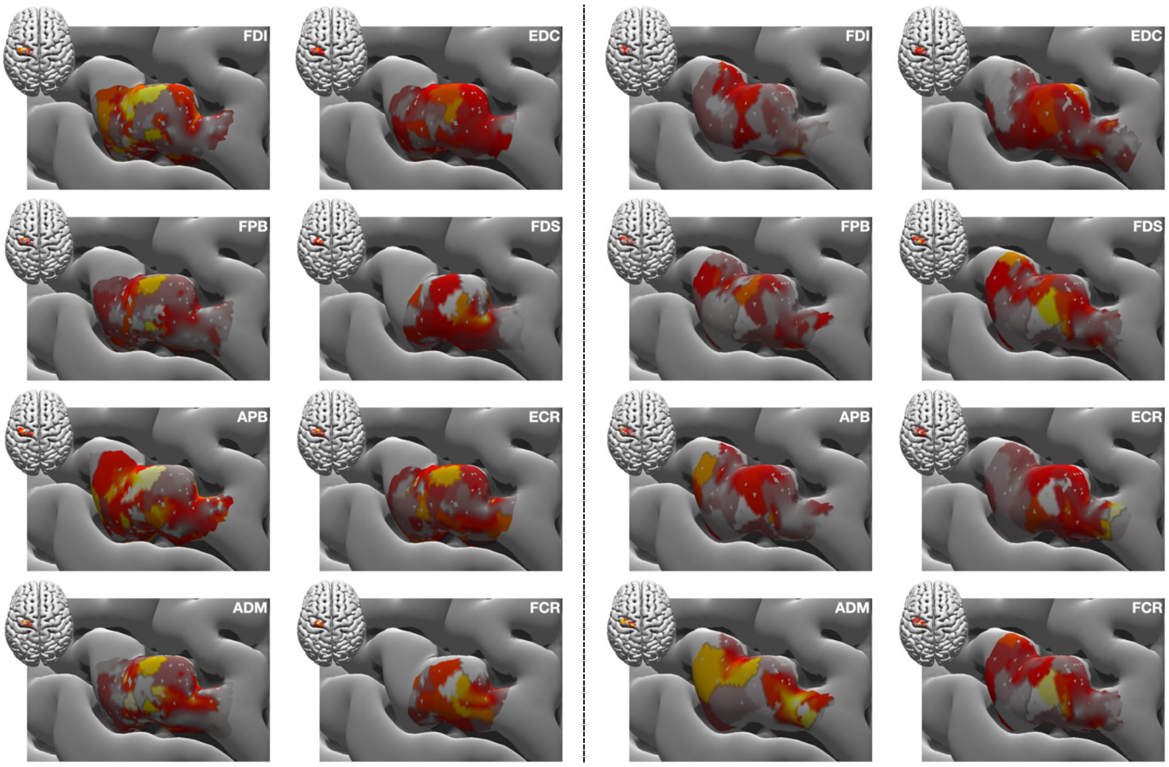

Figure S3.1 Excitability maps at 105%-RMT-FDI of two randomly selected participants; obviously the between-subject variability is large despite the warping to the MNI template.

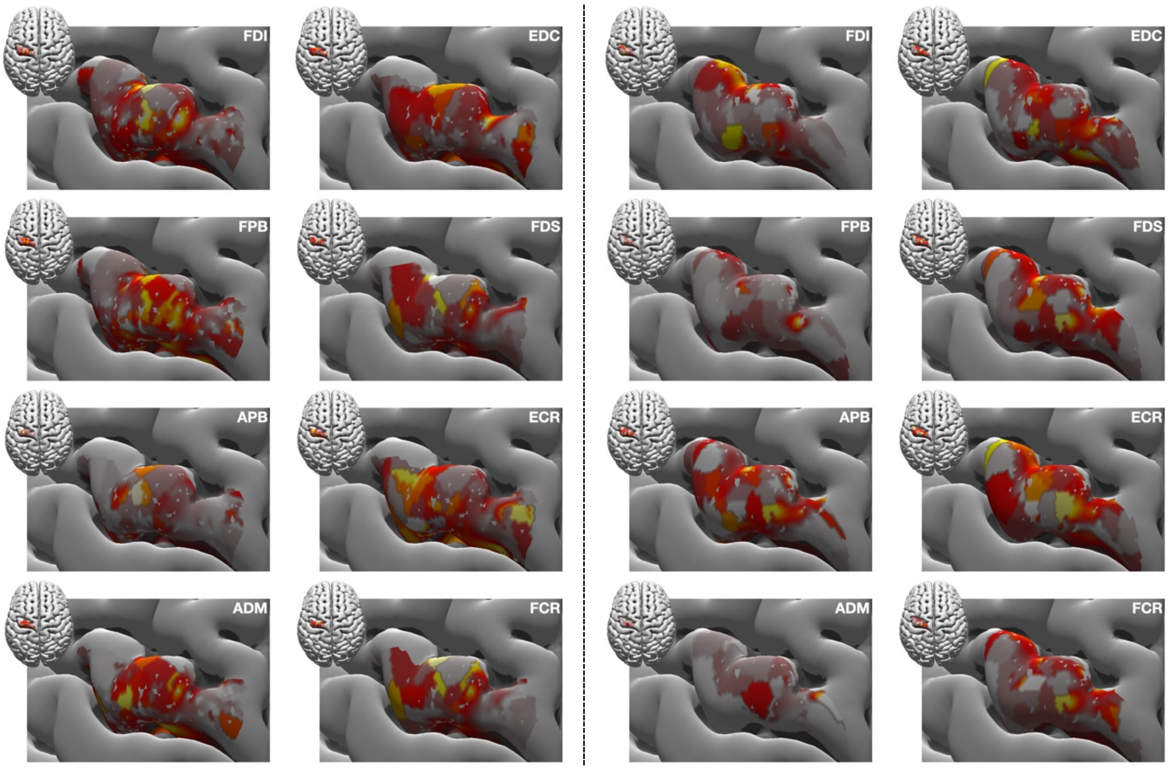

Figure S3.2 Identical to Figure S3.1 but for the 105%-RMT-EDC intensity.

S4. Centroid distributions in the posterior/anterior – lateral/medial plane

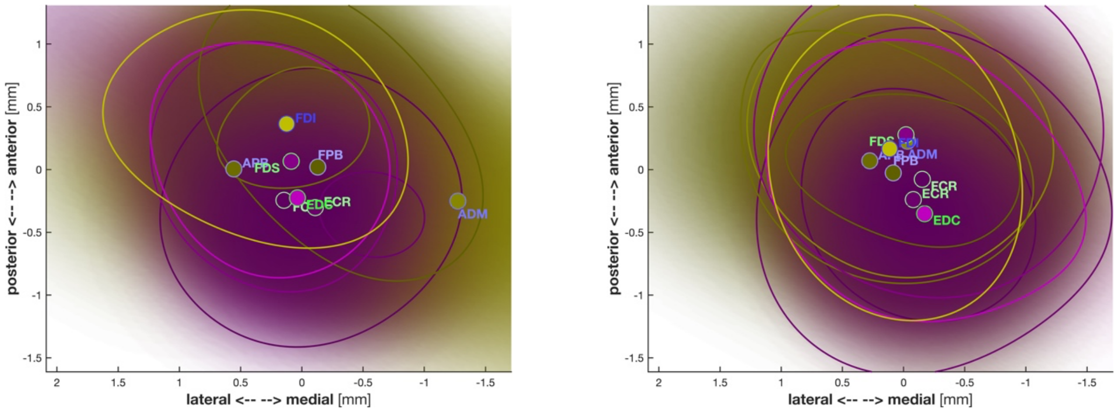

Figure S4.1 Centroids averaged for all participants at 105%-RMT-EDC and -FCR intensities (left and right panel, respectively); cf. Figure 6 in the main text. Centroids clearly differ for different intensities rendering them less appropriate to parametrise muscle-specific areas; cf. main text, Figure 6.

S5. Overlap statistics

Table S5.1 Statistics of overlaps and centroids between the three groups of muscle pairs. O_{kl} denotes the relative size of the pair-wise overlap and $C_{kl,x \dots z}$ the corresponding centroids in medial/lateral, anterior/posterior, and inferior/superior directions, respectively. Significant effects are in **bold.**

	Intensity		Group		Intensity × Group	
	F-value	*p*-value	F-value	*p*-value	F-value	*p*-value
O_{kl}	F(2,38)=6.246	**.005**	F(2,38)=208.862	**.000**	F(4,76)=5.326	**.001**
$C_{kl,x}$	F(2,26)=0.366	.697	F(2,26)=156.069	**.000**	F(4,52)=0.297	.771
$C_{kl,y}$	F(2,26)=0.052	.949	F(2,26)=377.245	**.000**	F(4,52)=0.281	.790
$C_{kl,z}$	F(2,26)=0.874	.429	F(2,26)=12176.4	**.000**	F(4,52)=0.972	.390

Table S5.2 Hand-hand pairs using the MNI template (constrained to M1). Significant effects in **bold**.

	Intensity		Pair		Intensity × Pair	
	F-value	*p*-value	F-value	*p*-value	F-value	*p*-value
O_{kl}	F(2,38)=4.979	**.012**	F(5,95)=4.021	**.034**	F(10,190)=0.422	.819
$C_{kl,x}$	F(2,26)=0.594	.559	F(5,65)=0.759	.468	F(10,130)=1.081	.377
$C_{kl,y}$	F(2,26)=0.282	.756	F(5,65)=4.023	**.012**	F(10,130)=0.557	.738
$C_{kl,z}$	F(2,26)=0.353	.706	F(5,65)=4.830	**.012**	F(10,130)=0.530	.696

Table S5.3 Same as Table S5.2 but for the forearm-forearm muscle pairs.

	Intensity		Pair		Intensity × Pair	
	F-value	*p*-value	F-value	*p*-value	F-value	*p*-value
O_{kl}	F(2,38)=3.768	**.032**	F(5,95)=3.870	**.016**	F(10,190)=0.433	.808
$C_{kl,x}$	F(2,26)=0.273	.763	F(5,65)=1.599	.223	F(10,130)=1.138	.346
$C_{kl,y}$	F(2,26)=0.142	.868	F(5,65)=3.162	**.043**	F(10,130)=0.771	.503
$C_{kl,z}$	F(2,26)=1.400	.265	F(5,65)=0.494	.670	F(10,130)=0.897	.467

Table S5.4 Same as Tables S5.2&3 but for the hand-forearm muscle pairs.

	Intensity		Pair		Intensity × Pair	
	F-value	*p*-value	F-value	*p*-value	F-value	*p*-value
O_{kl}	F(2,38)=6.987	**.003**	F(15,285)=1.859	.164	F(30,570)=0.668	.706
$C_{kl,x}$	F(2,26)=0.342	.714	F(15,195)=1.489	.229	F(30,390)=1.150	.343
$C_{kl,y}$	F(2,26)=0.045	.956	F(15,195)=4.214	**.005**	F(30,390)=1.062	.388
$C_{kl,z}$	F(2,26)=0.848	.440	F(15,195)=3.435	**.012**	F(30,390)=0.624	.672

S6. Statistics using subject-specific MRIs constrained to M1

Table S6 Statistics of the cortical muscle representation using subject-specific MRIs constrained to M1. W = area size, centroid components: C_x = medial/lateral, C_y = anterior/posterior, C_z = inferior/superior. The results agree qualitatively with the ones summarise in Table 1. Significant effects are in **bold**.

	Intensity		Muscle		Intensity × Muscle	
	F-value	*p*-value	F-value	*p*-value	F-value	*p*-value
C_x	F(2,32)=0.723	.493	F(7,112)=3.087	**.021**	F(14,224)=1.369	.235
C_y	F(2,32)=0.062	.940	F(7,112)=3.238	**.004**	F(14,224)=0.725	.748
C_z	F(2,32)=1.084	.334	F(7,112)=5.481	**.000**	F(14,224)=0.893	.506
W	F(2,30)=3.542	.072	F(7,105)=3.661	**.029**	F(14,210)=1.481	.246

Table S6.1 Same as Table S5.1 but using the subject-specific MRI constrained to M1.

	Intensity		Group		Intensity × Group	
	F-value	*p*-value	F-value	*p*-value	F-value	*p*-value
O_{kl}	F(2,38)=5.543	**.008**	F(2,38)=272.4530	**.000**	F(4,76)=4.058	**.013**
$C_{kl,x}$	F(2,28)=0.573	.570	F(2,28)=106.0840	**.000**	F(4,56)=1.461	.226
$C_{kl,y}$	F(2,28)=0.308	.738	F(2,28)=447.8270	**.000**	F(4,56)=0.407	.675
$C_{kl,z}$	F(2,28)=0.648	.470	F(2,28)=4434.2650	**.000**	F(4,56)=0.796	.444

Table S6.2 Same as Table S5.2 but using the subject-specific MRI constrained to M1.

	Intensity		Pair		Intensity × Pair	
	F-value	*p*-value	F-value	*p*-value	F-value	*p*-value
O_{kl}	F(2,38)=2.643	.084	F(5,95)=4.794	**.021**	F(10,190)=0.507	.746
$C_{kl,x}$	F(2,28)=1.557	.229	F(5,70)=1.851	.168	F(10,140)=0.298	.851
$C_{kl,y}$	F(2,28)=0.839	.443	F(5,70)=1.091	.358	F(10,140)=0.701	.552
$C_{kl,z}$	F(2,28)=0.241	.695	F(5,70)=5.027	**.023**	F(10,140)=0.730	.528

Table S6.3 Same as Table S5.3 but using the subject-specific MRI constrained to M1.

	Intensity		Pair		Intensity × Pair	
	F-value	*p*-value	F-value	*p*-value	F-value	*p*-value
O_{kl}	F(2,38)=3.602	**.037**	F(5,95)=5.774	**.001**	F(10,190)=0.659	.608
$C_{kl,x}$	F(2,28)=0.488	.619	F(5,70)=3.379	**.035**	F(10,140)=0.335	.789
$C_{kl,y}$	F(2,28)=0.057	.945	F(5,70)=4.064	**.011**	F(10,140)=0.710	.565
$C_{kl,z}$	F(2,28)=0.965	.362	F(5,70)=2.308	.090	F(10,140)=0.759	.573

Table S6.4 Same as Table S5.4 but using the subject-specific MRI constrained to M1.

	Intensity		Pair		Intensity × Pair	
	F-value	*p*-value	F-value	*p*-value	F-value	*p*-value
O_{kl}	F(2,38)=6.237	**.005**	F(15,285)=1.63	.199	F(30,570)=0.638	.719
$C_{kl,x}$	F(2,28)=0.623	.543	F(15,210)=2.642	.053	F(30,420)=0.446	.844
$C_{kl,y}$	F(2,28)=0.31	.736	F(15,210)=2.078	.103	F(30,420)=0.854	.513
$C_{kl,z}$	F(2,28)=0.674	.463	F(15,210)=3.902	**.013**	F(30,420)=0.874	.498

Tables S5.1…4 and S6.1…4 largely agree as expected given our statistical design (intensity, muscle, etc. are paired within subject). Qualitative differences are only incidental and occur solely in centroid components arguably due to difference in surface mesh resolution.

S7. Statistics using subject-specific MRIs without constraining to M1

Table S7 Same as Table S6 but without constraining the subject-specific MRIs to M1.

	Intensity		Muscle		Intensity × Muscle	
	F	*p*-value	F	*p*-value	F	*p*
C_x	F(2,34)=0.963	.392	F(7,119)=3.491	**.008**	F(14,238)=0.573	.726
C_y	F(2,34)=0.417	.663	F(7,119)=1.598	.143	F(14,238)=0.813	.576
C_z	F(2,34)=1.644	.208	F(7,119)=5.853	**.000**	F(14,238)=0.551	.776
W	F(2,32)=7.237	**.010**	F(7,112)=3.685	**.036**	F(14,224)=2.522	.080

Table S7.1 Same as Table S6.1 but without constraining the subject-specific MRIs to M1.

	Intensity		Group		Intensity × Group	
	F-value	*p*-value	F-value	*p*-value	F-value	*p*-value
O_{kl}	F(2,38)=4.735	**.015**	F(2,38)=388.614	**.000**	F(4,76)=3.147	**.043**
$C_{kl,x}$	F(2,32)=1.475	.244	F(2,32)=106.019	**.000**	F(4,64)=1.259	.299
$C_{kl,y}$	F(2,32)=0.009	.991	F(2,32)=374.543	**.000**	F(4,64)=0.441	.702
$C_{kl,z}$	F(2,32)=0.686	.511	F(2,32)=4271.004	**.000**	F(4,64)=0.969	.407

Table S7.2 Same as Table S6.2 but without constraining the subject-specific MRIs to M1.

	Intensity		Pair		Intensity × Pair	
	F-value	*p*-value	F-value	*p*-value	F-value	*p*-value
O_{kl}	F(2,38)=2.586	.107	F(5,95)=6.590	**.008**	F(10,190)=0.414	.840
$C_{kl,x}$	F(2,32)=0.780	.467	F(5,80)=1.694	.195	F(10,160)=0.760	.564
$C_{kl,y}$	F(2,32)=0.431	.654	F(5,80)=2.580	.069	F(10,160)=0.210	.929
$C_{kl,z}$	F(2,32)=0.593	.559	F(5,80)=8.773	**.001**	F(10,160)=0.528	.657

Table S7.3 Same as Table S6.3 but without constraining the subject-specific MRIs to M1.

	Intensity		Pair		Intensity × Pair	
	F-value	*p*-value	F-value	*p*-value	F-value	*p*-value
O_{kl}	F(2,38)=4.055	**.025**	F(5,95)=4.048	**.010**	F(10,190)=0.956	.440
$C_{kl,x}$	F(2,32)=1.493	.240	F(5,80)=1.289	.289	F(10,160)=0.874	.470
$C_{kl,y}$	F(2,32)=0.123	.885	F(5,80)=0.411	.655	F(10,160)=0.870	.486
$C_{kl,z}$	F(2,32)=0.811	.453	F(5,80)=2.157	.096	F(10,160)=0.666	.550

Table S7.4 Same as Table S6.4 but without constraining the subject-specific MRIs to M1.

	Intensity		Pair		Intensity × Pair	
	F-value	*p*-value	F-value	*p*-value	F-value	*p*-value
O_{kl}	F(2,38)=4.707	**.015**	F(15,285)=2.197	.112	F(30,570)=1.047	.404
$C_{kl,x}$	F(2,32)=1.602	.217	F(15,240)=1.651	.176	F(30,480)=0.541	.790
$C_{kl,y}$	F(2,32)=0.004	.996	F(15,240)=1.379	.252	F(30,480)=0.519	.768
$C_{kl,z}$	F(2,32)=0.758	.477	F(15,240)=4.510	**.004**	F(30,480)=0.553	.739

As expected, constraining stimulations to M1 has a major effect on the statistics across results both for the overlaps and the centroid positions.

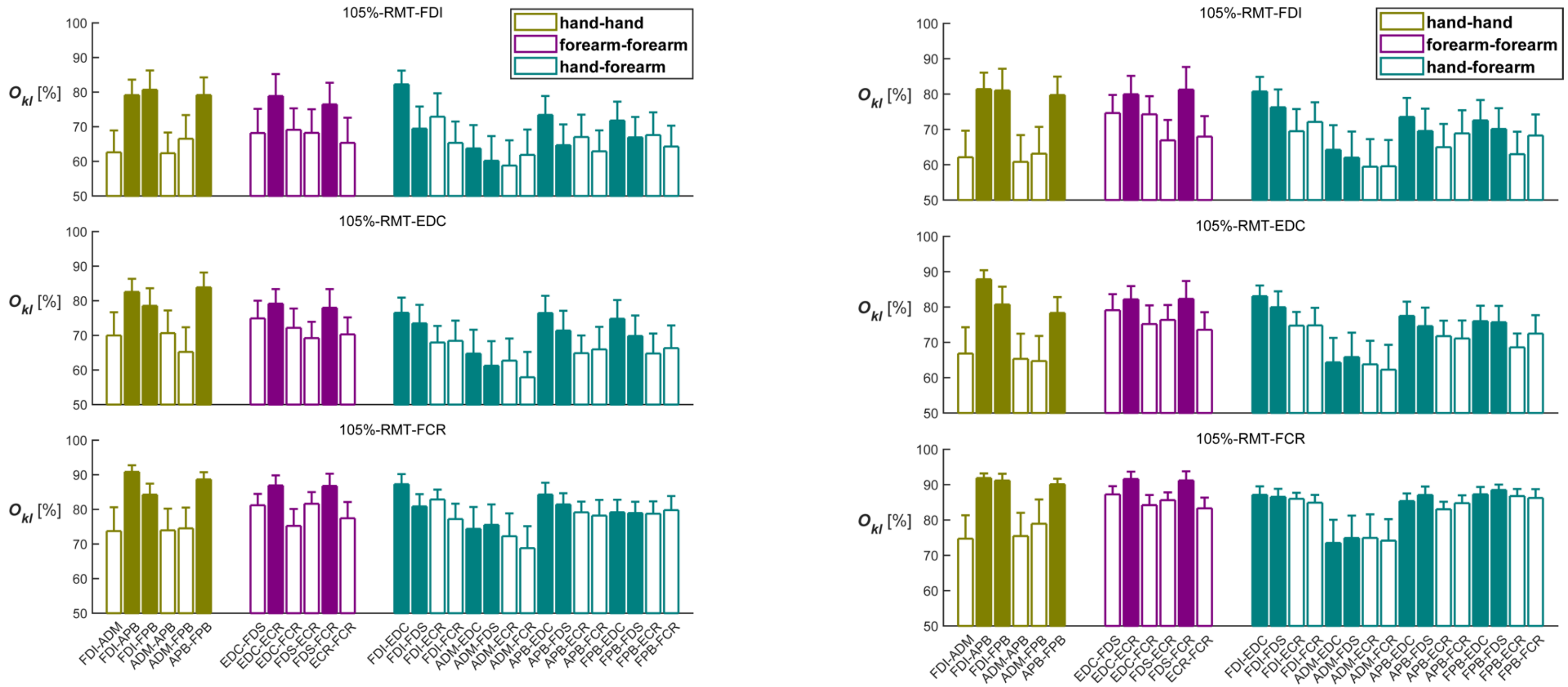

Figure S7.1 Overlap of all muscle pairs using subject-specific MRIs (left column: constrained to M1; right column: without constraint). We show the relative overlaps O_{kl} between muscle pairs expressed in percentage (of the union area) for the three intensities. The filled bars represent the muscle pairs with synergistic function, and the open bars represent the non-synergistic muscle pairs. Error bars represent the standard errors over participants. Both estimates agree qualitatively with the ones shown in Figure 7 of the main text, especially, when constraining to M1. This is expected due to the way we normalised O_{kl}. The minor quantitative differences stem from differences in resolution (triangularisation) of the cortex surfaces.

S8. Consistency over time of the elicited MEP amplitudes

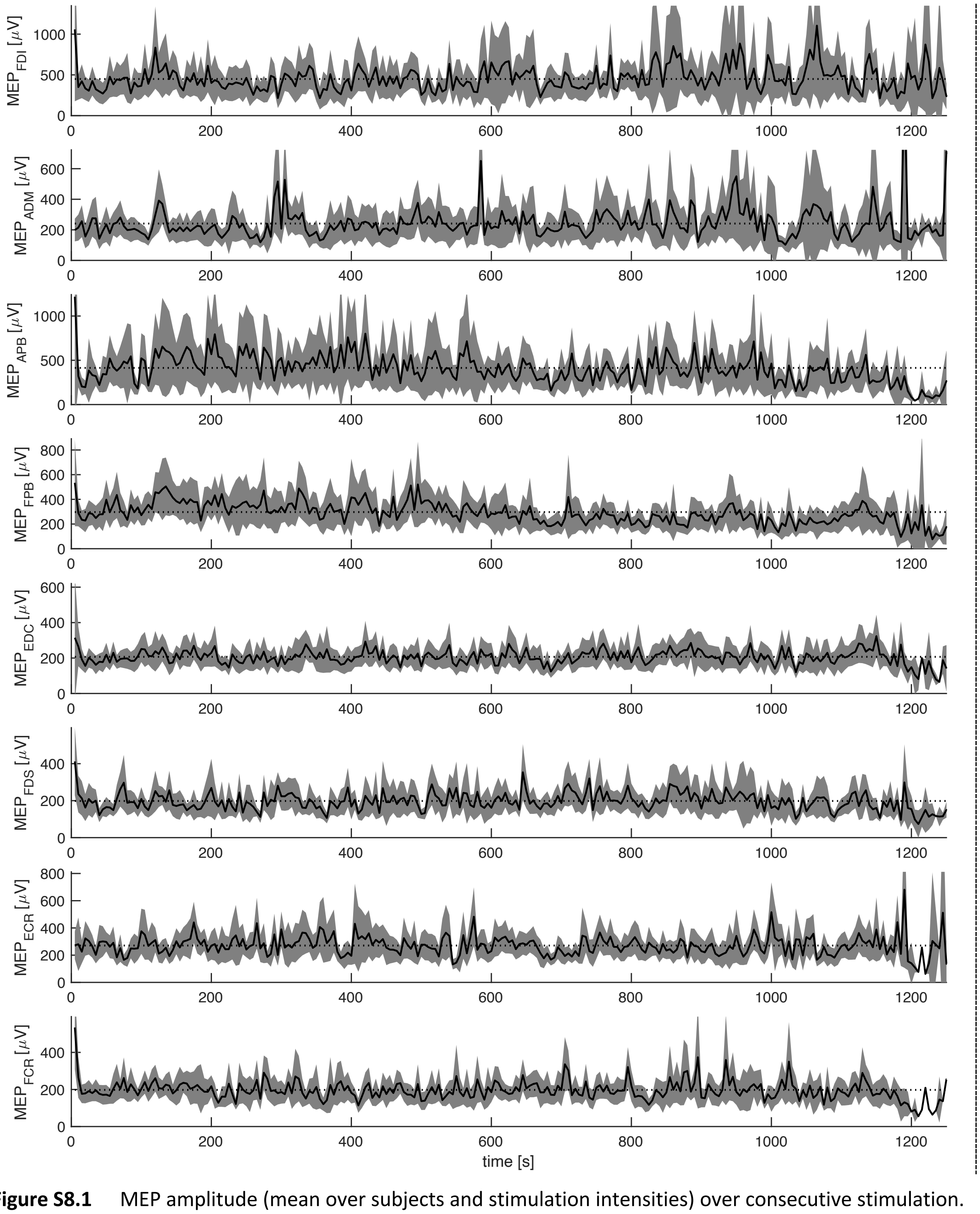

Figure S8.1 MEP amplitude (mean over subjects and stimulation intensities) over consecutive stimulation. TMS stimuli were separated by 5s inter-stimulus intervals. Here we included only the MEPs that were considered proper, non-MEP stimulations we set as missing values when computing the means – see body text and Jin, Bruijn, & Daffertshofer (2022). *Frontiers in Human Neuroscience*, *16*, 920538, for details. The grey patches indicate the 95% confidence interval, the dotted line indicated the mean value over all stimulations.
